# Comparative responsiveness and minimally important difference of Fatigue Symptom Inventory (FSI) scales and the FSI-3 in trials with cancer survivors

**DOI:** 10.1186/s41687-022-00488-1

**Published:** 2022-07-23

**Authors:** Catherine E. Mosher, Ekin Secinti, Shelley A. Johns, Kurt Kroenke, Laura Q. Rogers

**Affiliations:** 1 Department of Psychology, Indiana University-Purdue University Indianapolis, 402 North Blackford Street, LD 124, Indianapolis, IN 46202 USA; 2 Center for Health Services Research, Indiana University School of Medicine, Regenstrief Institute, 1101 W. 10th Street, Indianapolis, IN 46202 USA; 3 University of Alabama at Birmingham, 1720 2nd Avenue South, Birmingham, AL 35294 USA

**Keywords:** Cancer survivors, Fatigue Symptom Inventory, Minimally important difference, Psychometrics, Responsiveness to change

## Abstract

**Background:**

Fatigue is a highly prevalent and disabling symptom in cancer survivors. Although many measures have been developed to assess survivors’ fatigue, their ability to accurately capture change following intervention has rarely been assessed in post-treatment survivors. Ultra-brief fatigue measures are preferable in clinical practice but have limited evidence supporting their use with cancer survivors. We examined the psychometric properties of four Fatigue Symptom Inventory (FSI) measures, including the new FSI-3, in cancer survivors. Examined properties included responsiveness to change and minimally important differences (MIDs).

**Methods:**

We analyzed data from three randomized controlled trials with post-treatment cancer survivors (*N* = 328). Responsiveness to change was evaluated by comparing standardized response means for survivors who reported their fatigue as being better, the same, or worse at 2–3 months. Responsiveness to intervention was assessed via effect sizes, and MIDs were estimated by using several methods. We also computed area under the curve (AUC) values to assess FSI measures’ discriminative accuracy compared to an established cut-point.

**Results:**

All FSI measures differentiated survivors who reported improvement at 2–3 months from those with stable fatigue, but did not uniformly differentiate worsening fatigue from stable fatigue. Measures showed similar levels of responsiveness to intervention, and MIDs ranged from 0.29 to 2.20 across FSI measures. AUC analyses supported the measures’ ability to detect significant fatigue.

**Conclusions:**

Four FSI scales show similar responsiveness to change, and estimated MIDs can inform assessment of meaningful change in fatigue. The FSI-3 shows promise as an ultra-brief fatigue measure for survivors.

**Supplementary Information:**

The online version contains supplementary material available at 10.1186/s41687-022-00488-1.

## Plain English Summary

Fatigue is a highly prevalent and disabling symptom in cancer survivors. Although many measures have been developed to assess survivors’ fatigue, their ability to accurately capture change following intervention has rarely been assessed in post-treatment survivors. A minimally important difference in fatigue, or clinically meaningful change, also has rarely been documented for fatigue scales in studies of post-treatment survivors. Ultra-brief fatigue measures are preferable in clinical practice, but have limited evidence supporting their use with cancer survivors. This study aimed to address these gaps in the literature through analyses of Fatigue Symptom Inventory (FSI) data from three randomized behavioral intervention trials with post-treatment cancer survivors (*N* = 328). Four FSI measures (i.e., FSI total score, FSI severity and interference subscales, and the FSI-3) showed comparable ability to detect change in fatigue and responsiveness to intervention with cancer survivors. Estimated minimally important differences ranged from 0.29 to 2.20 across FSI measures and warrant further study to inform assessment of meaningful score change in research and clinical practice. Results support continued use of the FSI with cancer survivors and provide initial evidence for use of the ultra-brief FSI-3 measure.

**Supplementary Information:**

The online version contains supplementary material available at 10.1186/s41687-022-00488-1.

## Background

Fatigue is a highly prevalent, persistent, debilitating problem in cancer survivors [1]. Up to 40% of solid-tumor (e.g., breast, colorectal cancer) survivors experience moderate-to-severe fatigue in the first year after treatment [2, 3], and one third report this level of fatigue at 5 years post-diagnosis [3–5]. Fatigue often co-occurs with other disabling symptoms, such as pain, mood, and sleep problems [5–13], and has a substantial negative impact on activities [14–16]. Thus, reducing fatigue severity and interference with functioning is critical for improving survivors’ quality of life [12, 14, 16–18].

Many self-report measures have been developed to assess fatigue. The selection of a measure is guided by several considerations [19, 20]. These include the aspects of fatigue that one wishes to measure (e.g., severity, interference, time interval), respondent burden, the measure's clinical or research utility, and psychometric properties in the target population. The Fatigue Symptom Inventory (FSI) [21] is a 13-item self-report measure that assesses fatigue severity, fatigue frequency, and the interference of fatigue with activities, mood, and cognition. This measure has been extensively used with cancer survivors with strong evidence of construct validity and reliability [22]. In a review of fatigue measures for cancer populations, the FSI received the highest psychometric quality rating relative to other fatigue measures [23].

Multidimensional assessments of fatigue are recommended for clinical trials and other types of research [23]. In clinical settings, however, a brief measure may be preferable. Although 1-item fatigue measures have shown promise in cancer populations, they solely focus on fatigue severity and have inadequate evidence of validity or issues with responsiveness [20, 24]. A few measures of fatigue with 3 or 4 items have also been tested in cancer studies [25–28]. To date, these measures have been limited by their emphasis on one aspect of fatigue (e.g., physical sensations) [25] or lack of evidence of predictive validity and reliability [19, 26, 28]. Despite literature reviews on fatigue measurement [19, 20], there is no consensus regarding an optimal tool for briefly assessing fatigue in cancer populations. However, the International Society of Quality of Life Research has developed minimal standards for patient-centered outcomes, such as evidence of reliability, validity, responsiveness, and score interpretability [29].

An important psychometric property of fatigue measures is their responsiveness to change. Responsiveness includes the measures’ ability to accurately capture change following an intervention of known efficacy as well as change that is not the result of an intervention. In oncology research, clinical change in fatigue has primarily been evaluated in patients with advanced disease or those undergoing cancer treatment [30, 31]. In a review of FSI studies, effect sizes for responsiveness to change ranged from small to large in behavioral and disease treatment trials with cancer patients [22]. Standardized response means (SRMs), statistics that might better characterize the ability of the FSI to change over time, were unable to be computed. Another study found evidence of the FSI’s responsiveness to change by calculating effect sizes and SRMs for a trial targeting pain and depression during cancer treatment [32]. Further research is needed to document responsiveness to change for the FSI and other fatigue measures, especially in post-treatment survivors [19]. On average, fatigue tends to increase during cancer treatment and remit within 1 year following treatment [1, 33]. However, these averages mask substantial individual differences in survivors’ fatigue trajectories that warrant further assessment with responsive measures [1, 4].

Information on a minimally important difference (MID) in fatigue scores is another important psychometric property. An MID is the smallest difference in fatigue scores that the patient perceives as having positive or negative impact [34]. Perceived negative impact may lead the patient or clinician to consider a change in the patient’s fatigue management. Whereas an MID refers to change in fatigue that is meaningful to the patient irrespective of the context, a minimal clinically important difference (MCID) refers to change in patient-reported fatigue that leads to a change in clinical care [34]. MIDs for fatigue measures have substantially varied in studies of patients with advanced disease or those undergoing cancer treatment [35–38] and have yet to be evaluated among post-treatment survivors. As survivors recover from treatments, their MIDs may differ from those experiencing acute treatment side effects. MIDs for the FSI and its subscales have yet to be established.

Because different methods may produce somewhat different MID estimates, some experts recommend triangulating several approaches when estimating MIDs for a patient-reported outcome (PRO) [39, 40]. Distribution-based metrics include 0.2 to 0.5 standard deviations (SDs) or 1 to 2 standard errors of measurement (SEMs) [40–45]. However, these metrics are inferior to anchor-based methods and are instead best used as “supportive information for MID estimates from different anchor-based approaches and systematic reviews of the clinical trial literature” ^(p. 106)^ [39]. Common anchors include patient-rated global impression of change and comparison with absolute change on a legacy measure of the same domain. Whereas anchor-based methods have certain advantages [46], some PRO domains such as fatigue, pain, and some psychological symptoms may lack a criterion standard anchor. Moreover, the magnitude of an MID may vary depending upon whether one is measuring a difference or change at the level of an individual person vs. using aggregated individual-level data to compare differences between groups in research or clinical populations [47, 48]. To be considered meaningful, change within an individual may need to be larger than differences that are detected between groups [49].

This study aimed to determine the psychometric properties of four FSI measures in post-treatment cancer survivors enrolled in behavioral intervention trials. The measures were the FSI total score, FSI severity and interference subscales, and a new 3-item FSI (FSI-3). Examined psychometric properties included responsiveness to change (calculated via SRMs), responsiveness to intervention, and MID estimates. Additionally, area under the receiver operating curve (ROC) values were computed to estimate each FSI measure’s discriminative accuracy relative to an established cut-point, and a preliminary cut-point for the FSI-3 was determined based on these values. Cut-points are used in research and clinical practice to indicate need for further assessment or intervention.

## Methods

### Study samples

We analyzed data from three randomized controlled trials (RCTs) with post-primary treatment cancer survivors (*N* = 328) conducted between 2011 and 2015. Detailed methodology and results of these studies are published elsewhere [50–53]. These RCTs were approved by their respective institutional review boards (IRB approval numbers: Indiana University 1003-02B, 01206008951, Southern Illinois University School of Medicine 08–022, University of Alabama at Birmingham F121114008, University of Illinois Urbana-Champaign 09707), and all participants provided informed consent.

Briefly, sample 1 consisted of 35 cancer survivors participating in a pilot RCT comparing a 7-week Mindfulness-based Stress Reduction (MBSR) course for fatigue to a waitlist control condition (NCT01247532) [50]. Sample 2 consisted of 71 cancer survivors participating in a pilot RCT comparing the effects of an 8-week MBSR course vs. a psychoeducation/support group on fatigue (NCT01919853) [51]. For both trials, survivors were considered eligible if they were ≥ 18 years old with a non-metastatic cancer diagnosis; had not received cancer treatment in the past 3 months (other than endocrine therapy); reported persistent fatigue (≥ 2 months); and had clinically significant fatigue scores (FSI severity ≥ 4) at eligibility screening [50, 51]. Participants in both trials completed self-report questionnaires at baseline and approximately 2 months post-baseline. Given the similarities between samples 1 and 2, these data were combined for most analyses. Sample 3 consisted of 222 breast cancer survivors participating in an RCT comparing a 3-month physical activity behavior change intervention to usual care, with fatigue as a secondary outcome (NCT00929617) [52, 53]. For this trial, survivors were eligible if they were women between 18 and 70 years of age with a history of non-metastatic breast cancer; not currently receiving chemotherapy or radiation therapy; ≥ 2 months post-surgery; and participating in ≤ 30 min of vigorous or ≤ 60 min of moderate activity each week in the past 6 months [52, 53]. Our analyses focused on baseline and 3 months post-baseline assessments.

### Measures

The measures described below were administered in all three trials.

#### Fatigue Symptom Inventory (FSI)

The FSI is a 13-item fatigue self-report measure with evidence of reliability and validity in cancer populations [21, 54]. The FSI includes three subscales: severity, interference, and frequency of fatigue during the past week. FSI Severity is measured with four items using an 11-point scale (0 = *Not at all fatigued* to 10 = *As fatigued as I could be*). FSI Interference is measured with seven items using an 11-point scale (0 = *No interference* to 10 = *Extreme interference*). FSI frequency is measured with two items assessing the number of days (range = 0 to 7) and the extent of the day on average the respondents felt fatigued over the past week (0 = *None of the day* to 10 = *The entire day*). For each scale, the total score is the average of all items, with a higher score indicating worse fatigue.

We derived a 3-item fatigue measure (the FSI-3) that includes 1 severity item (average severity) and 2 interference items (interference with general activity and enjoyment of life) from the FSI. The FSI-3 uses the same scales and scoring (mean of the items) as the FSI. The FSI-3 was adapted from the 3-item PEG which is an ultra-brief version of the Brief Pain Inventory (BPI) [55]. The FSI and BPI items are identical except that fatigue is substituted for pain. The same severity and interference items have also recently been validated in the 3-item DEG scale for assessing dyspnea [56].

#### Short form-36 health survey vitality subscale (SF-36 vitality)

The SF-36 Vitality subscale (version 2) is a 4-item self-report measure assessing energy level during the past 4 weeks [57]. It is commonly used as a brief measure to assess fatigue in a variety of populations [58]. Scores are transformed into a scale ranging from 0 to 100, with higher scores indicating greater vitality. In prior research, survivors with scores less than 50 on the SF-36 Vitality measure showed biological and psychological/behavioral indicators of elevated fatigue compared to survivors with scores ≥ 50, supporting this cut-point’s validity [4, 33, 59–62]. Consistent with prior research [63], a more stringent cutoff (score ≤ 45) was used to indicate clinically meaningful fatigue. Given the measure’s established responsiveness to change [32, 64], it was used as an anchor when calculating responsiveness to change for fatigue measures in the current study.

### Data analysis

For most analyses, data from MBSR trials were combined and analyzed together, and data from the BEAT Cancer trial were analyzed separately. Analyses were performed using SPSS, version 25.0, R v.4.0.3 [65], and MedCalc statistical software [66]. Baseline and first follow-up data were used in the analyses (i.e., 2 months post-baseline in the MBSR trials and 3 months post-baseline in the BEAT Cancer trial). We calculated descriptive psychometrics (means, SDs, Cronbach’s αs), interscale correlations at baseline, and interscale correlations for change scores. Standard errors of measurement (SEMs) were computed by multiplying SDs by the square root of (1-Cronbach’s α). The coefficient of repeatability was calculated as 1.96 times SEM times the square root of 2 [47].

Responsiveness to change was examined by calculating SRMs for each fatigue measure (i.e., FSI severity, FSI interference, FSI-3, and FSI Total). SRMs are effect sizes calculated as the difference between mean scores within groups from baseline to post-intervention divided by the SD of the change score. SRMs of 0.2, 0.5, and 0.8 represent small, moderate, and large change, respectively [67]. We stratified SRMs by the following changes in SF-36 Vitality from baseline to follow-up: (1) Worse (i.e., fatigue increased by ≥ 1 SEM), (2) Same (i.e., fatigue remained about the same, change was < 1 SEM), and (3) Better (i.e., fatigue decreased by ≥ 1 SEM). We stratified the samples into worse-same-better groups using a legacy measure anchor, consistent with prior research [68, 69]. For each fatigue measure, pairwise *t*-tests were conducted comparing change scores in the Worse and Better categories with the change score in the Same category. The 95% CIs for each SRM were calculated via bias-corrected bootstrapping with 10,000 samples. Empirical cumulative distribution function plots were created to visually examine the distribution of change in fatigue scores by group (i.e., Worse, Same, Better).

Each measure’s responsiveness to intervention effects was examined by computing between-group treatment effect differences evaluated in two ways: (1) effect sizes, calculated as the difference between change score means of two independent groups divided by the pooled SD of baseline scores [70]; and (2) SRM, calculated as the difference between change score means of two independent groups divided by the SD of the pooled change score [71]. Given that MBSR trials included different control groups [50, 51], these between-group effect sizes were calculated for each trial separately. Effect sizes of 0.2, 0.5, and 0.8 represent small, moderate, and large change, respectively [72].

Four additional metrics were calculated for each FSI measure [43, 45]. First, we calculated 0.2 SD, 0.35 SD, and 0.5 SD for baseline FSI scores [73]. Second, we computed 1 SEM and 2 SEM for baseline FSI scores as lower and upper bounds of an MID. Third, we computed the difference in FSI score change from baseline to follow-up between the Better and Same SF-36 Vitality categories as a potential MID estimate. Fourth, we computed the coefficient of repeatability by multiplying 1.96 with the square root of 2 times SEM [47]. This metric is an estimate of the significance of individual change and is the minimal amount of change needed to be significant at *p* < 0.05 based on the reliable change index.

Area under the ROC curve (AUC) values were computed to estimate each FSI measure’s discriminative accuracy relative to the established cut-point for the SF-Vitality scale (≤ 45) at baseline. AUC values represent the probability of a measure correctly discriminating between survivors who had clinically significant fatigue and those who did not. An AUC value of 0.5 represents no ability to discriminate and 1.0 represents perfect discrimination.

To establish a preliminary cut-point for the FSI-3, AUC values and operating characteristics such as sensitivity (true positive rate), specificity (true negative rate), and Youden’s index (sensitivity + specificity  − 1) were examined. The ROC curve graphically represents the trade-off between sensitivity and specificity for the range of possible scores on the FSI-3 scale compared to the SF-Vitality criterion (≤ 45) at baseline. Given that the MBSR trials required elevated fatigue for eligibility, we only used the BEAT Cancer trial data to explore potential cut-points for the FSI-3.

## Results

### Patient characteristics

Additional file 2: Table 1 shows participants’ baseline characteristics. In the MBSR trials [50, 51], participants’ mean age was 57 years, and most were female (86%), non-Hispanic White (71%), and diagnosed with breast cancer (82%). In the BEAT Cancer trial [53], all participants were female breast cancer survivors, with an average age of 54 years. The majority were non-Hispanic White (82%).

### Descriptive psychometrics and interscale correlations

At baseline, all scale scores demonstrated good internal consistency reliability (*αs* = 0.74 to 0.95; Table 1). Within MBSR and BEAT Cancer trials, all FSI scales were strongly correlated at baseline. As expected, the SF-36 Vitality scale (for which a lower score represents worse fatigue) was negatively correlated with all FSI scales. A correlation ≥ 0.3 is one criterion that can used to support the use of an anchor [39, 40], a threshold clearly met by the SF-36 Vitality scale for which baseline correlations with the FSI scales ranged from − 0.44 to − 0.79 (correlations are negative because worse fatigue is represented by higher scores on the FSI scales vs. lower scores on the SF-36 Vitality scale). Additionally, change in FSI scores from baseline to follow-up showed moderate correlations with change in SF-36 Vitality scores (Additional file 3: Table 2).

**Table 1:** Descriptive psychometrics and correlations among the fatigue scales at baseline

Trials	Descriptive Psychometrics						Correlations			
	Mean	(SD)	Min	Max	α	SEM	FSI Total	FSI Severity	FSI Interference	FSI-3
MBSR (*N* = 106)										
FSI Total	5.06	(1.58)	0.54	8.00	0.93	0.43	–			
FSI Severity	5.25	(1.46)	1.00	8.50	0.81	0.63	0.81**	–		
FSI-Interference	4.79	(1.94)	0.00	8.71	0.91	0.59	0.97**	0.64**	–	
FSI-3	5.47	(1.83)	0.67	9.33	0.81	0.79	0.93**	0.75**	0.90**	–
SF-36 Vitality	32.41	(15.90)	0.00	75.00	0.74	8.05	− 0.57**	− 0.44**	− 0.53**	− 0.50**
BEAT (*N* = 222)										
FSI Total	3.85	(1.86)	0.92	8.92	0.95	0.40	–			
FSI Severity	4.58	(1.91)	1.00	9.25	0.89	0.63	0.90**	–		
FSI-Interference	3.38	(2.03)	1.00	9.29	0.93	0.52	0.96**	0.76**	–	
FSI-3	4.13	(2.19)	1.00	10.00	0.87	0.78	0.97**	0.87**	0.94**	–
SF-36 Vitality	51.23	(20.22)	0.00	87.50	0.84	8.08	− 0.79**	− 0.72**	− 0.75**	− 0.78**

^**^p < 0.01

MBSR = Mindfulness-based stress reduction. BEAT Cancer trial = Better Exercise Adherence after Treatment for Cancer trial. FSI = Fatigue Symptom Inventory. SF-36 Vitality = Short Form-36 Vitality subscale. SEM = Standard error of measurement, calculated as ${\text{SD}*}\sqrt {\left( {{{1 {-} \text{Cronbach}^{\prime}\text{s} \,\text{alpha}}}} \right)}$

### Responsiveness to change: standardized response means (SRMs)

Table 2 shows the change in FSI scores between baseline and follow-up (i.e., 2 months post-baseline in the MBSR trials and 3 months post-baseline in the BEAT Cancer trial) for each group (i.e., Worse, Same, or Better SF-36 Vitality scores). The *p *values denote the statistical significance of the change between the reference (i.e., Same) and other (i.e., Worse, Better) groups. Within trials, SRMs for fatigue measures yielded generally comparable results for each group. For the BEAT Cancer trial, all FSI measures significantly differentiated the Worse and Better groups from the Same group (SRMs: 0.69 to 0.95 and − 0.78 to − 0.96, respectively). For the MBSR trials, all FSI measures significantly differentiated the Better group from the Same group (SRMs:  − 0.88 to − 1.13). However, the measures did not significantly discriminate the Worse group from the Same group. Whereas all point estimates were negative (SRMs: − 0.24 to − 0.47), the confidence intervals included zero. Empirical cumulative distribution function plots showed that differentiation between groups (i.e., Better, Same, Worse) was better in the BEAT Cancer trial compared to the MBSR trials (Additional file 1: Figs. 1 and 2). Differentiation of the four versions of the FSI scales was similar within each trial.

**Table 2:** Change scores and standardized response means for fatigue measures

Fatigue measure Global category	MBSR trials (*N* = 106, Worse *n* = 11, Same *n* = 34, Better *n* = 59)				BEAT Cancer trial (*N* = 222, Worse *n* = 37, Same *n* = 89, Better *n* = 89)			
	Change score (SD)	*p *value	SRM	(95% CIs)	Change score (SD)	*p *value	SRM	(95% CIs)
FSI Total								
Worse	− 0.70 (1.57)	0.631	− 0.45	(− 1.16, 0.31)	1.22 (1.37)	< 0.001	0.89	(0.53, 1.20)
Same	− 0.98 (1.70)	–	− 0.58	(− 0.83, − 0.28)	0.05 (1.15)	–	0.05	(− 0.16, 0.26)
Better	− 1.96 (1.74)	0.010	− 1.13	(− 1.47, − 0.81)	− 1.19 (1.32)	< 0.001	− 0.90	(− 1.10, − 0.70)
FSI Severity								
Worse	− 0.55 (1.75)	0.759	− 0.31	(− 1.10, 0.37)	1.22 (1.29)	< 0.001	0.95	(0.63, 1.28)
Same	− 0.75 (1.95)	–	− 0.38	(− 0.70, − 0.08)	0.12 (1.37)	–	0.09	(− 0.13, 0.29)
Better	− 1.65 (1.88)	0.031	− 0.88	(− 1.18, − 0.57)	− 1.27 (1.62)	< 0.001	− 0.78	(− 0.99, − 0.57)
FSI Interference								
Worse	− 0.87 (1.85)	0.769	− 0.47	(− 1.28, 0.28)	1.19 (1.74)	< 0.001	0.69	(0.37, 0.99)
Same	− 1.06 (1.89)	–	− 0.56	(− 0.81, − 0.26)	0.03 (1.46)	–−	0.02	(− 0.19, 0.23)
Better	− 2.16 (2.01)	0.011	− 1.08	(− 1.40, − 0.74)	− 1.11 (1.42)	< 0.001	− 0.78	(− 0.99, − 0.57)
FSI-3								
Worse	− 0.45 (1.92)	0.332	− 0.24	(− 0.97, 0.51)	1.41 (1.66)	< 0.001	0.86	(0.47, 1.20)
Same	− 1.20 (2.25)	–	− 0.53	(− 0.82, − 0.21)	0.18 (1.34)	–	0.13	(− 0.08, 0.33)
Better	− 2.18 (2.05)	0.035	− 1.06	(− 1.44, − 0.64)	− 1.51 (1.56)	< 0.001	− 0.96	(− 1.18, − 0.76)

MBSR = Mindfulness-based stress reduction. BEAT Cancer trial = Better Exercise Adherence after Treatment for Cancer trial. SRM = Standardized Response Mean. FSI = Fatigue Symptom Inventory

“Worse” and “Better” are defined as ≥ 1 SEM decrease/increase from baseline to follow-up in the Short Form-36 Vitality subscale. Follow-ups for all trials occurred post-intervention (i.e., 2 months post-baseline for MBSR trials and 3 months post-baseline for the BEAT Cancer trial). *P*-values are from pairwise t-tests of change scores in the “Worse” or “Better” group compared with the change score in the “Same” group for each measure. Each SRM was calculated as the change score/SD of the change score. The 95% CIs for each SRM were calculated via bias-corrected bootstrapping with 10,000 samples using MedCalc software

### Responsiveness to intervention: between-group effect sizes

Table 3 shows the change in each measure by study condition for all trials and corresponding between-group effect sizes and SRMs. Of note, there were only modest differences between effect sizes and SRMs. Within each trial, all FSI scales showed similar levels of responsiveness to intervention. Across FSI and SF-36 Vitality measures, between-group effect sizes and SRMs were large for MBSR trial 1 and small to moderate for MBSR trial 2 and the BEAT Cancer trial.

**Table 3:** Change scores and between-group effect sizes and SRMs for intervention effects

Fatigue Measure	Change for Intervention Group (SD)	Change for Control Group (SD)	Between-group Effect Size	Between-group SRM
MBSR trial 1 (*N* = 35)				
FSI Total	2.31 (1.26)	− 0.31 (1.09)	1.51	2.22
FSI Severity	2.21 (1.41)	− 0.47 (1.20)	1.80	2.04
FSI-Interference	2.31 (1.55)	− 0.18 (1.46)	1.20	1.66
FSI-3	2.74 (1.48)	− 0.61 (1.80)	1.61	2.04
SF-36 Vitality	− 18.28 (15.47)	− 1.29 (12.56)	− 0.93	− 1.20
MBSR trial 2 (*N* = 71)				
FSI Total	1.92 (1.86)	1.58 (1.63)	0.23	0.20
FSI Severity	1.43 (2.01)	1.38 (1.92)	0.04	0.03
FSI-Interference	2.22 (2.07)	1.69 (1.94)	0.29	0.26
FSI-3	2.22 (2.05)	1.70 (2.03)	0.30	0.25
SF-36 Vitality	− 21.51 (19.29)	− 10.00 (13.49)	− 0.78	− 0.69
BEAT Cancer trial (*N* = 222)				
FSI Total	0.55 (1.49)	− 0.02 (1.53)	0.31	0.38
FSI Severity	0.49 (1.81)	0.05 (1.63)	0.23	0.25
FSI-Interference	0.58 (1.59)	− 0.09 (1.75)	0.33	0.40
FSI-3	0.65 (1.80)	− 0.02 (1.84)	0.30	0.37
SF-36 Vitality	− 11.61 (18.44)	0.21 (17.29)	− 0.58	− 0.66

MBSR = Mindfulness-based stress reduction. BEAT Cancer trial = Better Exercise Adherence after Treatment for Cancer trial. FSI = Fatigue Symptom Inventory. SF-36 Vitality = Short Form-36 Vitality subscale. Change = baseline score—post-intervention score. Between-group effect size = (Intervention group change—control group change)/ pooled baseline SD. Between-group SRM = (Intervention group change—control group change)/SD of pooled change score

### Additional psychometric values

Table 4 provides four psychometric values for the FSI scales in the MBSR and BEAT Cancer trials. The MID estimate using the global change anchor ranged from 0.90 to 1.69 on the 0 to 10 point FSI scales. The coefficient of repeatability ranged from 1.10 to 2.20. The two distribution-based metrics were somewhat lower, with 0.5 SD ranging from 0.73 to 1.10 and 2 SEM ranging from 0.79 to 1.59.

**Table 4:** Additional psychometric values for fatigue measures

	MBSR trials (*N* = 106)				BEAT Cancer trial (*N* = 222)				Average across trials			
	FSI Total	FSI Severity	FSI Interference	FSI-3	FSI Total	FSI Severity	FSI Interference	FSI-3	FSI Total	FSI Severity	FSI Interference	FSI-3
Global change (better vs same)	0.98	0.90	1.10	0.98	1.24	1.39	1.13	1.69	1.11	1.14	1.12	1.33
Coefficient of Repeatability	1.18	1.75	1.63	2.20	1.10	1.76	1.45	2.17	1.14	1.76	1.54	2.19
0.2 SD	0.32	0.29	0.39	0.37	0.37	0.38	0.41	0.44	0.34	0.34	0.40	0.40
0.35 SD	0.55	0.51	0.68	0.64	0.65	0.67	0.71	0.77	0.60	0.59	0.69	0.71
0.5 SD	0.79	0.73	0.97	0.92	0.93	0.95	1.01	1.10	0.86	0.84	0.99	1.01
1 SEM	0.43	0.63	0.59	0.79	0.40	0.63	0.52	0.78	0.41	0.63	0.55	0.79
2 SEM	0.85	1.27	1.18	1.59	0.79	1.27	1.04	1.56	0.82	1.27	1.11	1.58

MBSR = Mindfulness-based stress reduction. BEAT Cancer trial = Better Exercise Adherence after Treatment for Cancer trial. FSI = Fatigue Symptom Inventory

SD = Standard deviation of the baseline scores. SEM = Standard error of measurement of the baseline scores

“Better” refers to the group whose SF-36 Vitality scores increased by ≥ 1 SEM. “Same” refers to the group whose change in SF-36 Vitality scores was < 1 SEM. The best MID estimate was derived from triangulating three summary results (mean, median, and upper value of range excluding highest and lowest MID) and rounding to an integer value

### Area under the ROC (AUC)

Within trials, all FSI measures yielded similar AUC values for differentiating significant fatigue at baseline using an SF-36 Vitality score ≤ 45 as the criterion (Table 5). Most AUC values had acceptable to excellent discriminatory ability (range: 0.64–0.88, with 7 of 8 values being 0.74 or higher).

**Table 5:** Area under the ROC Curve (AUC) for fatigue measures

	MBSR trials (*N* = 106)		BEAT Cancer trial (*N* = 222)	
Fatigue Measure	AUC	SE	AUC	SE
FSI Total	0.742	0.071	0.883	0.023
FSI Severity	0.638	0.056	0.868	0.025
FSI-Interference	0.761	0.067	0.861	0.025
FSI-3	0.746	0.073	0.877	0.024

MBSR = Mindfulness-based stress reduction. BEAT Cancer trial = Better Exercise Adherence after Treatment for Cancer trial. FSI = Fatigue Symptom Inventory. AUC is the probability of correctly discriminating between survivors who reported significant fatigue based on the SF-36 Vitality score ≤ 45 and those who did not at baseline. SE = Standard error

For the FSI-3, visual inspection of the ROC curves and examination of operating characteristics across a range of scores suggested that a mean score ≥ 5 could be considered a preliminary cut-point. This cut-point optimizes the Youden index and takes into account the elbow of the ROC curve. It yielded a sensitivity of 0.77 and a specificity of 0.87 relative to the SF-36 Vitality score criterion (≤ 45). When examining the likelihood ratios (LRs) for FSI-3 score intervals, the 5–6 interval was the first interval with a LR > 1, suggesting that survivors with scores < 5 would be less likely to have clinically significant fatigue.

## Discussion

Across several trials with cancer survivors, four FSI measures, including the new FSI-3, showed evidence of internal consistency reliability (αs = 0.81 to 0.93) and convergent validity based on correlations with the SF-36 Vitality measure and among FSI measures. Furthermore, across trials, all FSI measures performed well in distinguishing improvement in fatigue from lack of change. However, the measures showed mixed ability across trials to distinguish worsening fatigue from lack of change. Additionally, responsiveness to behavioral interventions was similar across FSI measures, as were preliminary MID estimates. Finally, AUC analyses based on an established anchor supported the FSI measures’ ability to detect clinically significant fatigue.

SRMs were of large magnitude in survivors who improved over 2 or 3 months and differed significantly from SRMs in survivors with stable fatigue. SRMs were variable in survivors with worsening fatigue, and only differed significantly from SRMs in BEAT Cancer trial participants with stable fatigue. The small number of survivors with worsening fatigue in the MBSR trials (*n* = 11) may have contributed to the null findings. Another potential explanation is that the MBSR trials only enrolled survivors with significant fatigue at screening [50, 51]. Regression to the mean is more likely when initial fatigue scores are high. Finally, scales for other symptoms have also proven better at detecting improvement than worsening [42, 74, 75].

The FSI scales and SF-36 Vitality measure showed small to moderate change in response to a physical activity intervention with a usual care control and an MBSR intervention with an attention control. Conversely, these measures showed large change in the trial of MBSR versus a waitlist control. These effect sizes are typical for the literature on behavioral interventions for fatigue in cancer survivors [76, 77].

Our preliminary MID estimate using the global change anchor ranged from 0.90 to 1.69 on the 0 to 10 point FSI scales. Several limitations of this estimate should be noted. Rather than a comparison to a patient-reported global impression of change anchor (PGIC), our anchor defined change as ≥ 1 SEM change on the SF-36 vitality scale. This approach is used less commonly than the PGIC and, more importantly, did not allow for more granular assessments of change (e.g., small, moderate, large) captured by many PGIC scales. However, these are approximations and require further research in other samples and using additional MID approaches such as PGIC and other anchors [47, 48]. In the chronic illness literature, the magnitude of MIDs for fatigue measures has varied considerably based on the estimation method, patient population, and context [37].

Across FSI measures, AUC values ranged from 0.64 to 0.88, which represent moderate to strong differentiation between survivors with and without significant fatigue. Regarding the FSI-3, a preliminary cut-point ≥ 5 yielded optimal sensitivity and specificity relative to the widely used SF-36 Vitality subscale. Prior research has also supported a cut-point of 5 on a 0–10 scale for fatigue [78]. The FSI-3 addresses the need for a validated fatigue measure that briefly assesses both fatigue severity and interference in cancer populations. Across studies, the FSI-3 showed comparable psychometrics relative to lengthier FSI measures.

Limitations of the present analyses warrant mention. The samples primarily consisted of non-Hispanic White women who had completed primary treatment for early-stage breast cancer. Additionally, the FSI was originally developed in a sample that did not represent the racial and ethnic diversity of cancer survivors [21]. Generalizability of the findings to diverse cancer populations requires further research. Furthermore, the sample sizes for the MBSR trials were relatively small, which limited statistical power for detecting effects and analyzing specific racial or ethnic groups. The SF-36 Vitality subscale was the only anchor used in analyses of responsiveness to change and AUC values and has a slightly different time frame than the FSI (past 4 weeks vs. 1 week). There is no criterion standard for testing the responsiveness of fatigue measures. Although the SF-36 Vitality subscale is one of the most established measures with evidence of responsiveness to change [32], it also relies on patient self-report. Other indices of improvement (e.g., activity engagement) would strengthen our findings. In addition, although a 3-category approach (i.e., Worse, Same, or Better SF-36 Vitality scores) has commonly been used to determine MIDs and converges with distribution-based methods [68, 69], it may result in overestimation of MIDs. Finally, the psychometric properties of the FSI-3, including a cut-point and its sensitivity to change, warrant replication. Test–retest reliability should also be assessed.

## Conclusions

In summary, examined FSI measures had comparable responsiveness to change and preliminary estimates of MID in cancer survivors. Our results strongly support continued use of the FSI with survivors and provide initial evidence for use of the FSI-3. As fatigue is a top concern of survivors [79], its rigorous assessment is an important first step in optimizing quality of life. Examining the generalizability of our findings to non-cancer populations is also important given the substantial prevalence of fatigue across many medical and psychological conditions as well as the potential benefits of measurement-based care for optimal fatigue management.

## Supplementary Information

**Additional file 1.** **Supplemental figures 1 and 2.** Empirical cumulative distribution function plots.

**Additional file 2.** **Supplemental table 1.** Patient characteristics.

**Additional file 3.** **Supplemental table 2.** Correlations of change scores among the fatigue scales.

## Data Availability

The datasets used and/or analyzed during this study are available from the corresponding author on reasonable request.

## Abbreviations

AUC: Area under the curve
BPI: Brief pain inventory
FSI: Fatigue Symptom Inventory
LR: Likelihood ratio
MBSR: Mindfulness-based stress reduction
MID: Minimally important difference
RCT: Randomized controlled trial
ROC: Receiver operating curve
SEM: Standard error of measurement
SF-36: Short-form 36
SRM: Standardized response mean
