# Supplementary material for: Comparative responsiveness and minimally important difference of Fatigue Symptom Inventory (FSI) scales and the FSI-3 in trials with cancer survivors
Source: J Patient Rep Outcomes. 2022 Jul 23;6:82. doi: 10.1186/s41687-022-00488-1 (PMC9308850; doi:10.1186/s41687-022-00488-1)
Supplement: Supplementary file 1 — Additional file 1. Supplemental figures 1 and 2. Empirical cumulative distribution function plots. [file 41687_2022_488_MOESM1_ESM.docx]

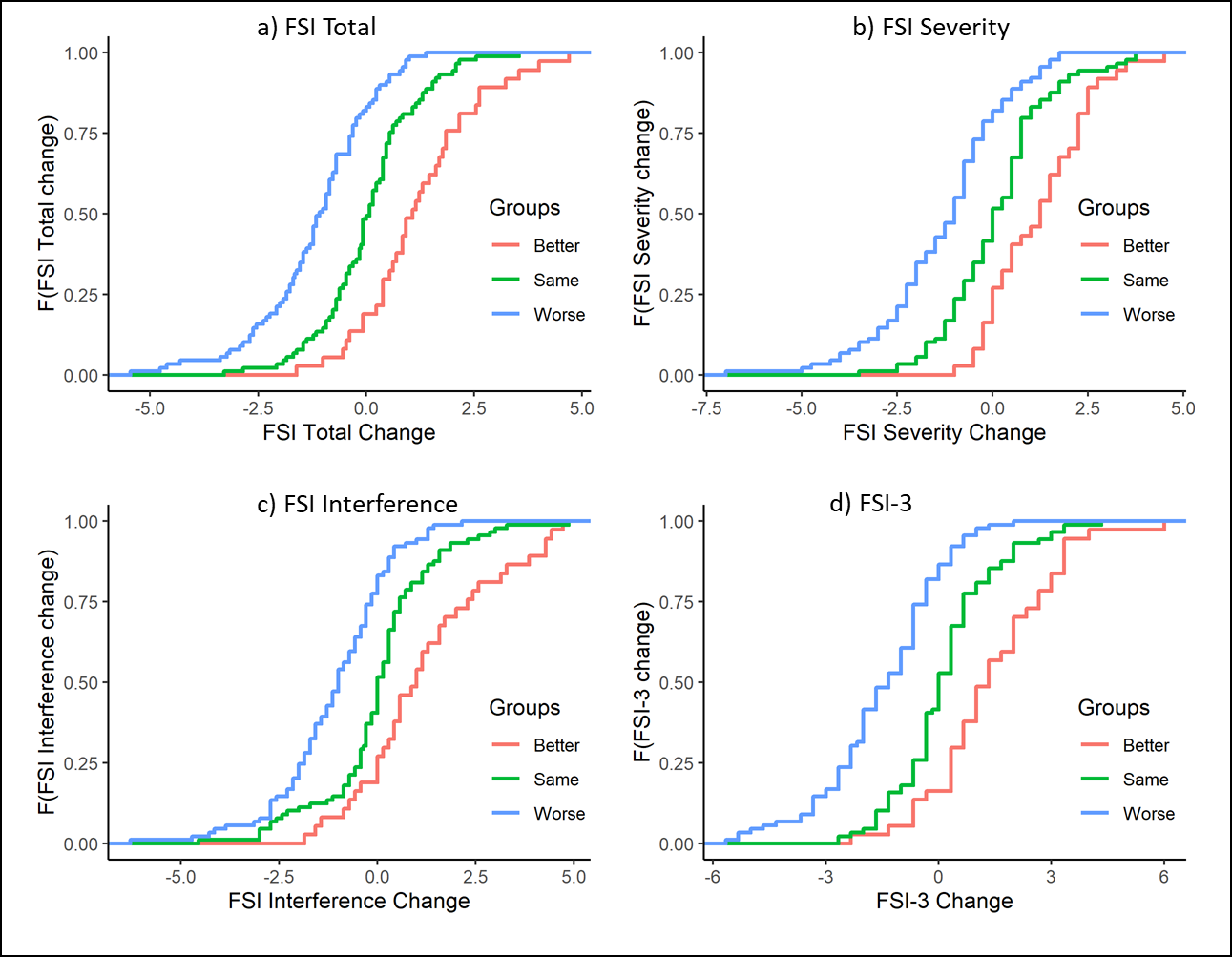


**Supplemental Figure 1.** Empirical cumulative distribution function plots depicting the distribution of change in fatigue scores from baseline to post-intervention by group (i.e., Worse, Same, Better) for the BEAT Cancer Trial.

FSI: Fatigue Symptom Inventory.


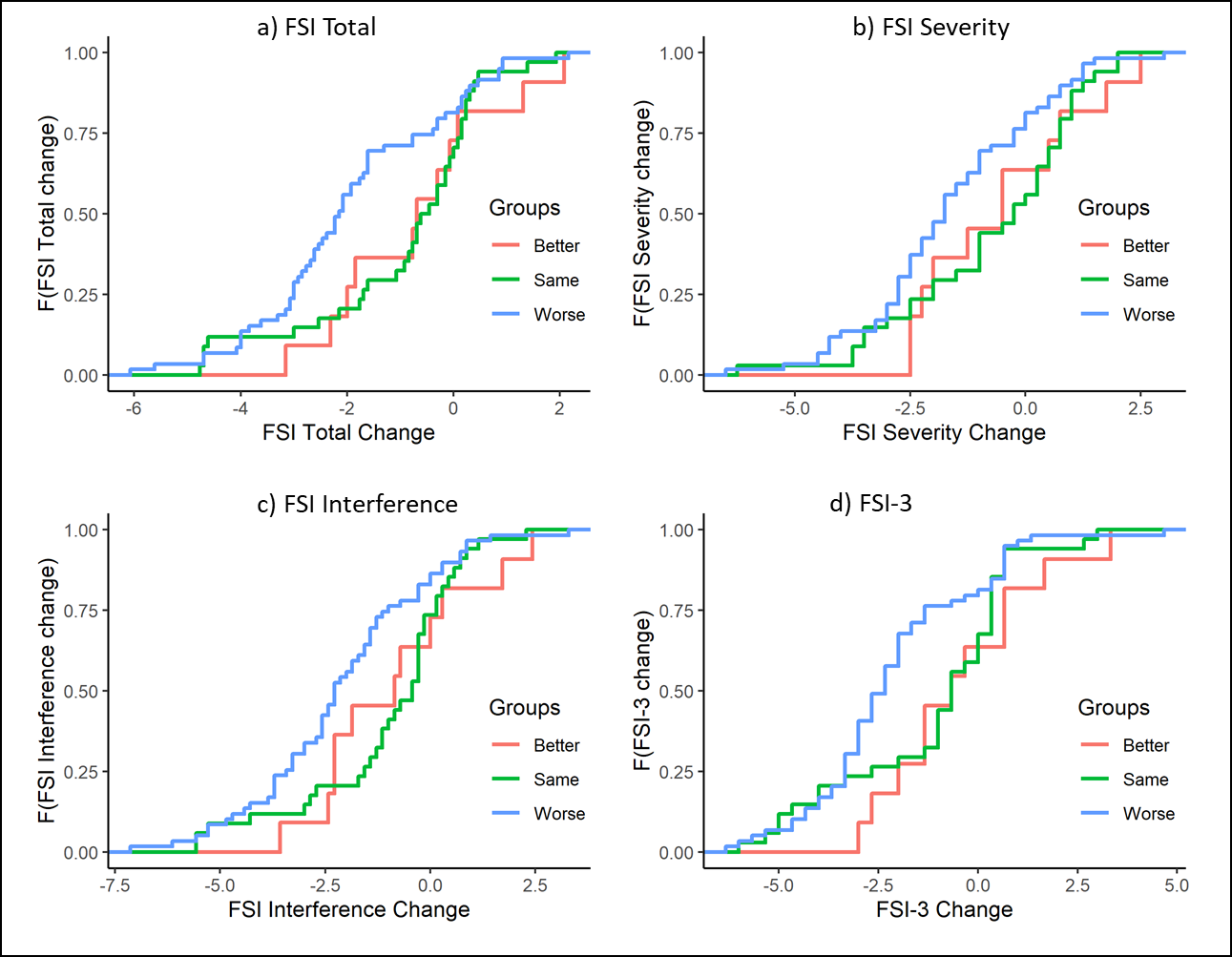


**Supplemental Figure 2.** Empirical cumulative distribution function plots depicting the distribution of change in fatigue scores from baseline to post-intervention by group (i.e., Worse, Same, Better) for the MBSR Trials.

FSI: Fatigue Symptom Inventory.
