# Supplementary material for: Comparative responsiveness and minimally important difference of Fatigue Symptom Inventory (FSI) scales and the FSI-3 in trials with cancer survivors
Source: J Patient Rep Outcomes. 2022 Jul 23;6:82. doi: 10.1186/s41687-022-00488-1 (PMC9308850; doi:10.1186/s41687-022-00488-1)
Supplement: Supplementary file 2 — Additional file 2. Supplemental table 1. Patient characteristics. [file 41687_2022_488_MOESM2_ESM.docx]

**Supplemental Table 1.** Patient characteristics

| Patient Characteristic | MBSR trials  (*N =* 106) | | BEAT Cancer trial  (*N =* 222) | |
| --- | --- | --- | --- | --- |
| **Demographics** |  | |  | |
| Age, mean (*SD*) | 56.86 | (10.6) | 54.41 | (8.5) |
| Female, % | 97 | (85.5%) | 222 | (100.0%) |
| Race and ethnicity: |  |  |  |  |
| Non-Hispanic White, % | 75 | (70.8%) | 183 | (82.4%) |
| Non-Hispanic Black/African American, % | 22 | (20.8%) | 24 | (10.8%) |
| Hispanic, % | 2 | (1.9%) | 3 | (1.4%) |
| Other, % | 7 | (6.6%) | 12 | (5.4%) |
| Married/Partnered, % | 60 | (56.6%) | 158 | (71.2%) |
| College Education, % | 57 | (53.8%) | 127 | (57.2%) |
| Employed, % | 54 | (50.9%) | 157 | (70.7%) |
|  |  |  |  |  |
| **Medical factors** |  |  |  |  |
| Breast cancer % | 87 | (82.1%) | 222 | (100.0%) |
| Chemotherapy % | 55 | (51.9%) | 128 | (57.7%) |
| Radiation % | 52 | (49.1%) | 151 | (68.0%) |
| Surgery % | 70 | (66.0%) | 222 | (100.0%) |
| Hormone therapy* % |  |  | 108 | (48.65%) |
| *Hormone therapy information was only available for the BEAT Cancer trial.  MBSR = Mindfulness-based stress reduction. BEAT Cancer trial = Better Exercise Adherence after Treatment for Cancer trial. | | | | |
