# Supplementary material for: Comparative responsiveness and minimally important difference of Fatigue Symptom Inventory (FSI) scales and the FSI-3 in trials with cancer survivors
Source: J Patient Rep Outcomes. 2022 Jul 23;6:82. doi: 10.1186/s41687-022-00488-1 (PMC9308850; doi:10.1186/s41687-022-00488-1)
Supplement: Supplementary file 3 — Additional file 3. Supplemental table 2. Correlations of change scores among the fatigue scales. [file 41687_2022_488_MOESM3_ESM.docx]

**Supplemental Table 2.** Correlations of change scores among the fatigue scales

| **Trials** | **Correlations of Change Scores** | | | | |
| --- | --- | --- | --- | --- | --- |
|  | **FSI**  **Total** | **FSI**  **Severity** | **FSI**  **Interference** | **FSI-3** |  |
| **MBSR** (*N =* 106) |  |  |  |  |  |
| FSI Total | -- |  |  |  |  |
| FSI Severity | .83** | -- |  |  |  |
| FSI-Interference | .95** | .64** | -- |  |  |
| FSI-3 | .94** | .79** | .90** | -- |  |
| SF-36 Vitality (continuous) | -.40** | -.33** | -.36** | -.38** |  |
| SF-36 Vitality (categorical) | -.30** | -.25* | -.30** | -.30** |  |
| **BEAT** (*N =* 222) |  |  |  |  |  |
| FSI Total | -- |  |  |  |  |
| FSI Severity | .83** | -- |  |  |  |
| FSI-Interference | .93** | .60** | -- |  |  |
| FSI-3 | .93** | .79** | .88** | -- |  |
| SF-36 Vitality (continuous) | -.68** | -.59** | -.60** | -.67** |  |
| SF-36 Vitality (categorical) | -.58** | -.54** | -.50** | -.61** |  |
| ***p* <.01.  MBSR = Mindfulness-based stress reduction. BEAT Cancer trial = Better Exercise Adherence after Treatment for Cancer trial. FSI = Fatigue Symptom Inventory. SF-36 Vitality = Short Form-36 Vitality subscale. SF-36 Vitality (categorical) is coded as -1 = Worse, 0 = Same, 1 = Better.  Changes in scores were calculated from baseline to post-intervention. | | | | |  |
